# Effects of historical legacies on soil nematode communities are mediated by contemporary environmental conditions

**DOI:** 10.1002/ece3.6406

**Published:** 2020-05-27

**Authors:** Xianping Li, Xiaoyun Chen, Huimin Zhu, Zhuhong Ren, Jiaguo Jiao, Feng Hu, Manqiang Liu

**Affiliations:** ^1^ Soil Ecology Lab College of Resources and Environmental Sciences Nanjing Agricultural University Nanjing China; ^2^ Jiangsu Key Laboratory for Solid Organic Waste Utilization Jiangsu Collaborative Innovation Center for Solid Organic Waste Resource Utilization Nanjing China

**Keywords:** climate change, current climate, historical imprint, soil biodiversity, soil fauna

## Abstract

Both contemporary and historical factors are documented to be crucial in regulating species diversity and distribution. Soil fauna contribute substantially to global biodiversity and ecosystem functioning, while it is unclear whether and to what extent historical factors shape their diversity patterns. Here, we used soil nematodes as a model organism to test historical effects on soil fauna and to investigate the relative importance of climatic, soil, and historical factors. Based on nematode distribution data in 16 natural sites at a large scale (ranging from 22 to 40°N) in mainland China, we conducted elastic net regression model to test the effects of climatic (e.g., mean and seasonality of temperature/precipitation), soil (e.g., soil carbon, nitrogen, and pH), and historical (e.g., temperature/precipitation anomaly and the velocity of the change since the Last Glacial Maximum) variables on nematode genus richness and Shannon's diversity. Additionally, variation partitioning was used to determine the contribution of the three predictor sets to the explanation of both Jaccard and Bray–Curtis community dissimilarity. We found that climate generally explained more variations in both diversity and composition than soil and historical predictors in our samples. We also showed that although historical factors (e.g., temperature change velocity) were correlated with nematode diversity and composition, the pure effects of these historical factors were negligible. In other words, the historical effects were commonly represented by their interactions with current climatic and soil factors within our selected sites. Our results indicated that contemporary factors, especially climate, may outperform historical factors in regulating soil nematode diversity patterns at large scales.

## Graphical abstract

Although historical factors (e.g., temperature change velocity) were correlated with nematode diversity and composition, the pure effects of these historical factors were negligible. The historical effects on soil nematode diversity patterns were commonly represented by their interactions with current climatic and soil factors at large scales.

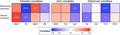

## 1 INTRODUCTION

Understanding species diversity patterns and their potential determinants is a fundamental topic in ecology and biogeography (Fine, 2015; Macarthur, 1965). Numerous studies have shown that both current factors (e.g., climate and topography) and historical factors (e.g., past environmental changes) play unique roles in regulating the diversity and distribution of those aboveground macroorganisms, including plants (Kissling et al., 2012; Svenning & Skov, 2007), amphibians (Araújo et al., 2008), reptiles (Araújo et al., 2008), birds (Hawkins & Porter, 2003; Qian, 2008), and mammals (Davies, Buckley, Grenyer, & Gittleman, 2011; Hawkins & Porter, 2003). However, it is still unclear whether and how historical factors affect the diversity belowground (Delgado‐Baquerizo et al., 2017; Mathieu & Davies, 2014) despite soil biodiversity contributing substantially to both global biodiversity and ecosystem functioning (Bardgett & van der Putten, 2014; Wardle et al., 2004).

Given that the number of studies in soil biogeography (mainly focusing on soil bacteria and fungi) has begun to increase (Bahram et al., 2018; Delgado‐Baquerizo et al., 2018; Fierer & Jackson, 2006; Tedersoo et al., 2014; Thompson et al., 2017; Wu, Ayres, Bardgett, Wall, & Garey, 2011), detailed assessments of historical imprints (e.g., effects of long‐term climate changes) on soil biodiversity are still lacking (excluding those phylogeographic studies about historical legacies in intraspecific genetic diversity; Delgado‐Baquerizo et al., 2017; Ji et al., 2019). Additionally, the limited studies that focused on soil microorganisms have shown different results. For example, Delgado‐Baquerizo et al. (2017) found that paleoclimate explained more variation in the bacterial richness and composition than the current climate, while Ji et al. (2019) suggested that the fungal richness and composition were primarily or comparably influenced by the contemporary environment. Studies on soil fauna are generally lagging behind their aboveground counterparts and belowground microorganisms. Through comparing the diversity among sites that experienced glaciation or not, Mathieu and Davies (2014) suggested that past glaciations contributed to the latitudinal gradients in earthworm diversity, and Fiera, Habel, Kunz, and Ulrich (2017) showed that the phylogenetic diversity and structure of Collembolan communities were also influenced by glaciations in Europe. These findings demonstrated that soil organisms could also be regulated by historical factors.

Soil nematodes are a widespread and species‐rich group that perform important functions in soil ecosystems, making them valuable biological indicators of soil quality and soil health (Bongers & Ferris, 1999; Yeates, 2003). Some studies have demonstrated the crucial role of contemporary climates in the development and maintenance of soil nematode diversity (Chen et al., 2015; Nielsen et al., 2014; Song et al., 2017). For example, Nielsen et al. (2014) found that nematode composition was strongly related to mean annual rainfall and temperature at a global scale, and Chen et al. (2015) demonstrated that the variation in soil nematodes was primarily explained by precipitation at a regional scale. Additionally, it is well known that nematode diversity, abundance, and composition are determined by soil conditions, such as soil carbon and pH, which have been well documented by various studies at both local and large scales (Chen et al., 2015; Liu et al., 2016; Quist et al., 2019; van den Hoogen et al., 2019; Wu et al., 2011). However, we know nothing about the effects of those historical factors like long‐term climate changes on soil nematodes. Given soil nematodes generally have low dispersal ability and are sensitive to environmental changes (Bongers & Ferris, 1999; Yeates, 2003), for a given site if there were severe long‐term climate changes, part or all of nematodes may go regionally extinct resulting in low diversity and a simple community. Thus, we suppose that there could also be obvious historical imprints on the current diversity and composition of soil nematodes.

To test whether and to what extent historical factors shape soil nematode diversity patterns, we investigated the distribution of soil free‐living nematodes in mainland China over a wide spatial scale and gathered the main climatic, soil, and historical information of these sites. Then, the variation in diversity and composition of soil nematodes was partitioned into the components of independent and shared effects among the three predictor sets (i.e., climatic, soil, and historical variables). We hypothesize that long‐term historical factors would have negative effects on soil nematode communities, and their effects are independent from those current climatic and soil factors.

## 2 MATERIALS AND METHODS

### 2.1 Nematode distribution data

To obtain a panorama of the diversity patterns of soil nematodes at large scales, we obtained soil samples from 16 natural sites in mainland China (Figure S1). We selected the sites by maximizing the geographical and environmental gradients considering both accessibility and affordability (Table S1 and Figure S1; Li et al., 2020). Three plots were selected within each site, and each plot (approximately 20 m^2^) was at least 100 m apart from each other. For each plot, five soil cores (diameter: 3.5 cm; depth: 0–10 cm) were randomly collected and mixed. A total of 48 soil samples were collected from August to October 2016 during the plant growth stage to minimize the seasonal effect in our study (Li et al., 2020). Although the sample size is relatively small, the sampling procedures were highly comparable among these sites. After transportation to the laboratory, 100 g soil was weighed for nematode extraction using a modified Baermann method followed by sugar centrifugal flotation (Liu et al., 2008). All nematodes in a sample were counted first; then, approximately 150 randomly chosen individuals per sample were identified to genus level with a light microscope (Bongers, 1988). All plots were measured separately, but the values were then averaged to the site level.

### 2.2 Climatic, soil, and historical variables

We used three categories of variables to assess the potential drivers of soil nematode diversity, namely, current climate, soil properties, and historical factors. Four climatic variables, including the mean values of annual temperature (MAT, °C), annual precipitation (MAP, mm), temperature seasonality (TS), and precipitation seasonality (PS), were extracted from WorldClim (http://www.worldclim.org/) at a resolution of 30 arc‐second (approximately 1 km at the equator; Hijmans, Cameron, Parra, Jones, & Jarvis, 2005). We used three key variables to describe the soil conditions for each site. Soil organic carbon (SOC; g/kg) and total nitrogen (TN; g/kg) were measured using a C/N analyzer. Soil pH was measured using a soil water suspension (1:2.5 weight/volume) with a pH meter. Additionally, four historical variables that characterized climate changes since the Last Glacial Maximum (LGM; approximately 21,000 years before present) were adopted, that is, temperature/precipitation anomaly and velocity of temperature/precipitation change. We selected the LGM here because climatic changes since the LGM has been documented to affect the diversity patterns of various taxonomic groups (Araújo et al., 2008; Delgado‐Baquerizo et al., 2017; Qian, 2008; Sandel et al., 2011; Svenning & Skov, 2007). The historical MAT at the LGM was obtained at a spatial resolution of 2.5 arc‐minutes (the highest resolution available for the LGM) from WorldClim, and the mean values were calculated from two models, CCSM and MIROC. The current MAT for the calculations of historical variables was also obtained from WorldClim at the same resolution. Temperature anomaly (TS) was measured as the difference in MAT between the present and the LGM (Sandel et al., 2011). We calculated the velocity of change in temperature (TCV) using the method adopted by Sandel et al. (2011), which integrates both temporal and spatial gradients in temperature. Briefly, we first obtained the temporal gradient, that is, temperature anomaly. The spatial gradient was calculated as the slope of the spatial MAT gradient based on current MAT by considering the four nearest neighbors of each cell. Then, the velocity was calculated through dividing the temporal gradient by the spatial gradient. The precipitation anomaly (PA) and precipitation change velocity (PCV) were acquired using the same methods based on precipitation‐related variables. TCV and PCV were log‐transformed to improve normality for further analyses. Although the WorldClim data were based on modelling, we assumed that the differences in the values would reflect the actual differences among sites at a large scale used here (Hijmans et al., 2005).

### 2.3 Data analysis

We first assessed the relationships between single predictor and nematode genus richness or Shannon's diversity ( $-\sum_{i=1}^{S}p_{i}\ln p_{i}$ , *S* is the number of genera, *p_i_* is the proportion of genus *i*) using bivariate correlation. As the variables in different sets (i.e., climatic, soil, and historical predictors sets) are highly correlated (Araújo et al., 2008; Hawkins & Porter, 2003; Table S2) and the sample size is not large enough, it would be difficult to discriminate the effect of each predictor on nematode diversity using a multiple linear regression model. Nevertheless, as the multicollinearity will not materially affect the performance of the model (e.g., adjusted *R*‐squared; Kutner, Nachtsheim, Neter, & Li, 2005), and we are actually interested in whether each predictor set plays a unique role on nematode diversity, thus, we conducted elastic net (EN) regression model to test the effects of these predictor sets. EN is a regularization and variable selection method that can reduce overfitting efficiently by shrinking the coefficient estimates (Zou & Hastie, 2005). The EN has two turning parameters, alpha (a value controlling the relative strength of two types of regularizations, namely, ridge and lasso) and lambda (a value defining the amount of shrinkage). The best values of these parameters were determined with fivefold cross‐validation through selecting the alpha and lambda that minimize the root mean squared error. The adjusted *R*‐squared (*R* ^2^_adj_) was used to characterize the performance of the selected model with the formula 1 – (1 − *R* ^2^) × (*n*–1)/(*n *– *p *– 1) (*R* ^2^ is the proportion of variance explained, *n* is sample size, and *p* is the number of predictors). This procedure was repeated 100 times to reduce the instability in the result of a single cross‐validation, and the mean *R* ^2^_adj_ value was calculated. To measure the relative effects of the three predictor sets on nematode diversity, we first calculated the *R* ^2^_adj_ values for all the combinations of the three predictor sets; then, the *R* ^2^_adj_ values were used to partition the independent and shared effects of these predictor sets, namely, pure effects of climatic, soil, and historical variables and the overlaps between two and among three of them (Araújo et al., 2008; Svenning & Skov, 2005). A permutation approach was used to test the significance of each fraction. Briefly, null distributions of *R* ^2^_adj_ values for the seven fractions were generated through randomly permuting the dependent variable 999 times. A fraction was considered to be statistically significant when its observed value is greater than the 95% quantile of the null distribution values. As we did not detect spatial autocorrelation in the residuals of the selected models for all the combinations of the predictor sets using Moran's I statistic (all the *p*‐values based on permutation test > .05), the spatial predictors (e.g., the geographic coordinates of the sites) were not included in the models, let alone the use of a spatial regression model.

Nonmetric multidimensional scaling (NMDS) was conducted to visualize the community dissimilarity between assemblages using two widely used indices, namely incidence‐based Jaccard index ((*b* + *c*)/(*a* + *b* + *c*), *a* is the number of genera present in both sites, and *b* and *c* are the number of genera that are unique to each site) and abundance‐based Bray–Curtis index ( $\sum_{i=1}^{S}\left|x_{\mathrm{ij}}-x_{\mathrm{ik}}\right|/\left(\sum_{i=1}^{S}x_{\mathrm{ij}}+\sum_{i=1}^{S}x_{\mathrm{ik}}\right)$ , *x_ij_* and *x_ik_* are the abundance of genus *i* on site *j* and site *k,* respectively). To assess the relationship between community composition and the predictor variables, we fitted the environmental variables onto the ordination with function *envfit* in package vegan (Oksanen et al., 2019), and the significance of the variables was assessed based on 999 permutations. The relationship between community dissimilarity and spatial distance was evaluated by a Mantel test with 999 permutations. Variation partitioning in the distance‐based approach was used to determine the contribution of the three predictor sets to the explanation of community dissimilarity (Tuomisto, Ruokolainen, & Yli‐Halla, 2003). The differences in climatic, soil, and historical predictors between sites were characterized using the Euclidean distance on all the principal components obtained from the principal component analyses for each predictor set separately. The total dissimilarity was partitioned into independent and shared effects of the three predictor sets. Here, the *R* ^2^_adj_ was used to assess the fit of the models. The significance of each fraction was also tested using a permutation test (*n* = 999). All analyses were conducted using R 3.3.0 (R Core Team, 2016).

## 3 RESULTS

### 3.1 Nematode diversity

**FIGURE 1:**
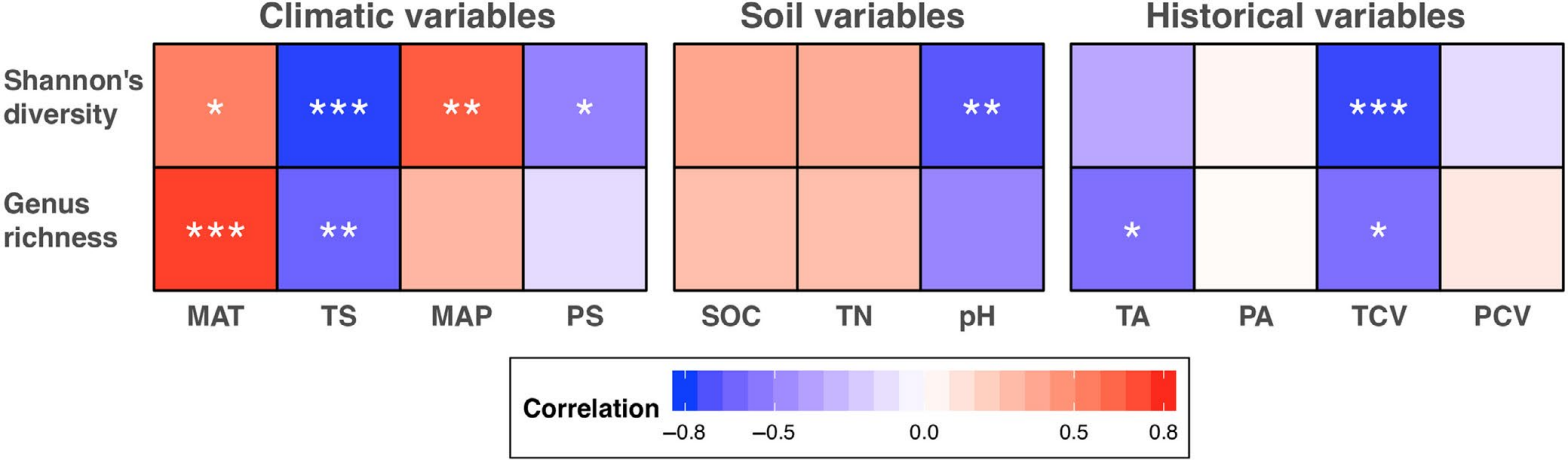
Correlations between climatic, soil, or historical variables and soil nematode diversity (genus richness and Shannon's diversity). The number of stars shows the significance (*p < .05; **p < .01; and ***p < .001). MAP, annual precipitation; MAT, annual mean temperature; PA, precipitation anomaly; PCV, precipitation change velocity; PS, precipitation seasonality; SOC, soil organic carbon; TA, temperature anomaly; TCV: temperature change velocity; TN, total nitrogen; TS, temperature seasonality

We recorded a total of 64 nematode genera from 35,935 individuals. The number of genera ranged from 17 to 41 with a mean value of 30 among the sites (Tables S1 and S3). We found that the genus richness and Shannon's diversity both significantly declined with latitude (*r* = −.64 and −.77; all *p* < .05). Among the climatic variables, annual mean temperature was positively and temperature seasonality was negatively associated with the two diversity metrics. Shannon's diversity was also correlated with annual precipitation and precipitation seasonality (all *p* < .05; Figure 1). In addition, the abundance‐based Shannon's diversity was significantly negatively associated with soil pH (Figure 1). We also found long‐term climate change (i.e., temperature change velocity) was negatively correlated with these diversity measures, and temperature anomaly showed a negative association with nematode richness (Figure 1).

**FIGURE 2:**
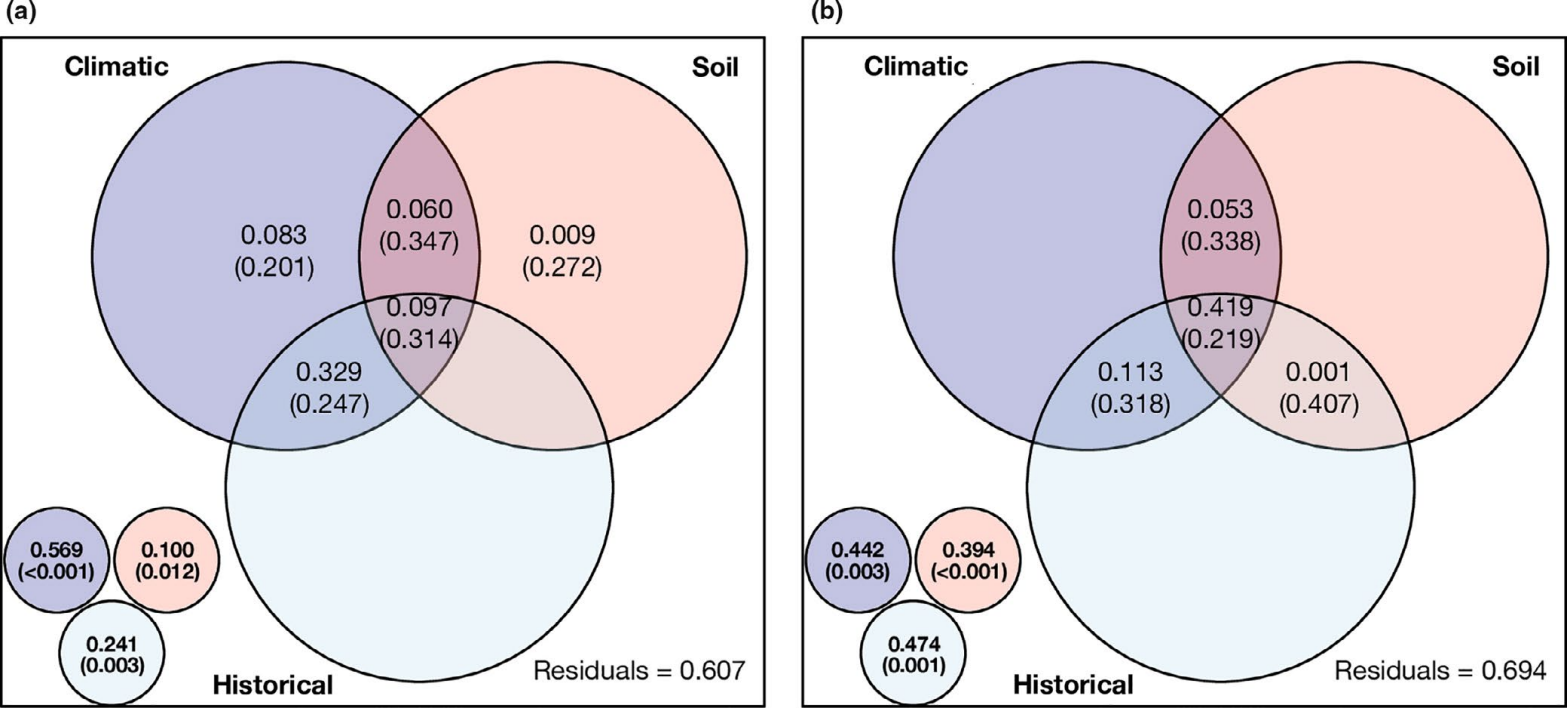
Contribution of different combinations of climatic, soil, and historical factors to the explanation of soil nematode genus richness (a) and Shannon's diversity (b). The insets in the bottom left show the total variation explained by each predictor set. The *p*‐value from permutation tests are listed in parentheses for each fraction, and significant values (*p* < .05) are shown in bold. Please note the sum of variation explained by multiple individual fractions of a predictor set is not equal to the total variation explained by this predictor set because some fractions can explain negligible variation (i.e., adjusted *R*‐squared < 0; data not shown)

The results from EN models showed that the total effect (including independent and shared effects) of climatic, soil, or historical factors was significant for genus richness and Shannon's diversity, while there were no significant pure effects detected (Figure 2). Additionally, although the shared effects between and among the three predictor sets contributed substantially to the explained variation for the two diversity metrics, the *R* ^2^_adj_ values of these fractions were not significantly different from the simulated null values (permutation test: all *p* > .05; Figure 2). Moreover, we also found slightly more variation was explained by the model of incidence‐based genus richness than the model of abundance‐based Shannon's diversity (39.3% vs. 30.6%; Figure 2).

### 3.2 Nematode composition

**FIGURE 3:**
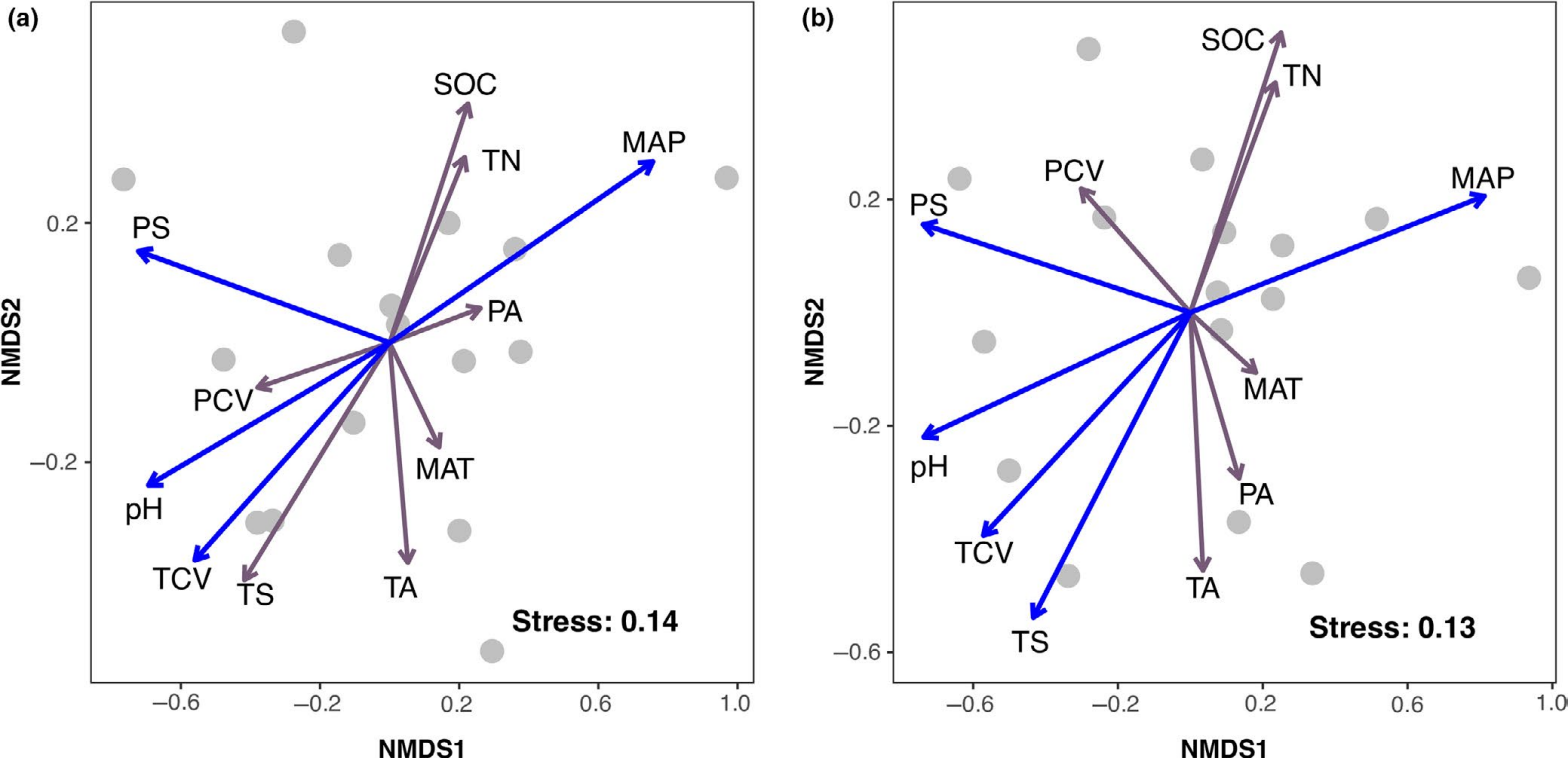
Nonmetric multidimensional scaling plots based on Jaccard (a) and Bray–Curtis (b) dissimilarities of soil nematode communities. Variables in blue show significant relationship with nematode composition (permutation test: *p* < .05). Variable abbreviations as in Figure 1

**FIGURE 4:**
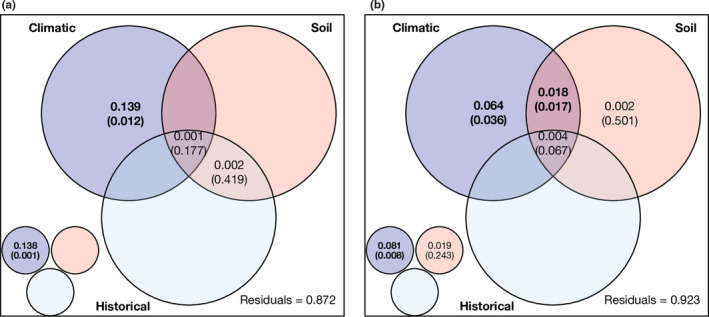
Contribution of different combinations of differences (i.e., Euclidean distances) in climatic, soil, and historical factors to the explanation of soil nematode community dissimilarity (a: Jaccard index; b: Bray–Curtis index). See Figure 2 for a detailed description of the figures

The NMDS plots demonstrated that mean annual precipitation, precipitation seasonality, soil pH, and historical temperature change velocity were significantly correlated with the composition of nematode communities (Figure 3). Additionally, temperature seasonality was significantly associated with abundance‐based community composition too. The community dissimilarity was not correlated with spatial distance (Mantel *r* = .091 for Jaccard index and 0.107 for Bray–Curtis index; both *p* > .05; Figure S2). After decomposing the variation in community dissimilarity into climatic, soil, and historical differences, we found that these predictor sets explained relatively little of the total variation in community dissimilarity (<15%; Figure 4). The total and pure climatic effects were consistently significant for Jaccard and Bray–Curtis dissimilarities (permutation test: *p* < .05; Figure 4). Only the fraction shared between climatic and soil predictor sets was found to have a significant effect on Bray–Curtis dissimilarity (Figure 4). Other fractions (e.g., total and pure effects of soil or historical predictor set) generally showed negligible and nonsignificant effects on community dissimilarity.

## 4 DISCUSSION

It is well known that the patterns of species diversity are likely mediated by both contemporary and historical processes, while the effects of historical factors on soil biodiversity have not been fully clarified, not to mention the exploration of the redundancy and complementarity between the contemporary and historical processes. Based on our current data, we found that apart from the well‐documented climatic and soil factors, soil nematode diversity, and composition are also associated with historical factors like long‐term climate changes. However, the results also suggested that the effects of historical factors are shared with the climatic and soil factors, indicating that contemporary environmental conditions could be treated as the main determinants of soil nematode communities in our samples at a large spatial scale.

Numerous studies have demonstrated that biodiversity declines with increasing latitude at large scales for aboveground organisms (Hillebrand, 2004; Pianka, 1966), while the generality of this rule in soil nematodes is still under debate (Kerfahi et al., 2016; Porazinska et al., 2010; van den Hoogen et al., 2019; Wu, Chen, & Zhang, 2016; Wu et al., 2011; Zhang, Pennings, Li, & Wu, 2019). Our results support that soil nematode diversity also has a latitudinal trend, which is higher diversity at lower than higher latitudes. This trend could be caused by the fact that the covaried climatic and soil variables are latitude‐dependent (Figure S3). Indeed, significant associations between those key climatic (e.g., the mean value and seasonality of temperature/precipitation) and soil (e.g., pH) factors and soil nematode diversity were supported by the current and earlier studies (Franco et al., 2019; Nielsen et al., 2014; Wu et al., 2011). Additionally, we also noticed that the abundance‐based diversity (i.e., Shannon's diversity) is significantly associated with these precipitation variables (i.e., mean annual precipitation and precipitation seasonality), emphasizing the crucial direct and indirect roles of precipitation in regulating nematode population dynamics. This finding also calls attention to the necessity of incorporating changes in both temperature and precipitation for evaluating the effects of future climate changes on soil nematodes. The generally weak effects of soil predictors on nematode diversity could be partly due to climate being more important for diversity at large scales than at local scales (Hawkins et al., 2003), and study with limited sample size may fail to detect the potential effects of those variables that changed locally and considerably across time and space (van den Hoogen et al., 2019).

Interestingly, we found that sites with higher temperature change velocity have lower nematode genus richness and Shannon's diversity. Although the high extinction rates and low levels of recolonization in those sites might contribute to lower diversity, as documented in aboveground macrofaunal groups (Araújo et al., 2008; Dynesius & Jansson, 2000; Sandel et al., 2011), this phenomenon can also be caused by the fact that climate change velocity is highly correlated with other climatic and soil factors (Table S2). Thus, understanding the relative importance of historical factors compared to climatic and soil factors is crucial to verify the real effect of historical factors on nematodes. Through decomposing the variation in nematode diversity into the independent and shared effects of the three sets of predictor variables, we found that the shared effects between and among these predictor sets are substantial, especially for the overlaps between climatic and other (soil and historical) predictor sets, indicating current climates can be seen as the most comprehensive determinants of nematode diversity in our samples. While this finding is inconsistent with a global study, which found effects of soil characteristics overwhelmed those of climate in driving nematode abundance (van den Hoogen et al., 2019). This may result from we focused on different response variables (abundance vs. diversity) and quantified the relative importance in different approaches (van den Hoogen et al. ranked the variables individually, while we treated each predictor set as a whole). Nevertheless, our study suggested that the historical effects on nematode diversity can be potentially represented by current environmental conditions, although significant correlations between historical factors and current diversity were recorded in our data. In addition, we found that the compositions of nematodes are correlated with climatic (i.e., mean annual precipitation and precipitation seasonality), soil (i.e., pH), and historical (i.e., temperature change velocity) factors. Moreover, our results suggested that both total and pure effects of current climate are significant determinants of nematode community dissimilarity, while no historical effects were detected. Although the climatic and soil conditions are well known to be crucial determinants of the composition of soil nematode communities (Nielsen et al., 2014; Wu et al., 2011), the relative effects of these variables and the historical factors are only partitioned in this study. More studies in different habitats and with large samples are needed to verify these findings.

Unlike studies in aboveground macrofauna including amphibians, reptiles, birds, and mammals (Araújo et al., 2008; Davies et al., 2011; Hawkins & Porter, 2003), we did not find significant independent effects of historical factors on soil nematode diversity and composition within our samples. This absence could be due to (a) China did not experience severe glaciation during the Pleistocene in comparison to Europe and North America (Qian & Ricklefs, 2000); (b) animals with small body size and large population size, such as nematodes, might be resistant to adverse climatic changes (Gardner, Peters, Kearney, Joseph, & Heinsohn, 2011; Williams, Shoo, Isaac, Hoffmann, & Langham, 2008); (c) the highly heterogeneous soil habitat may allow soil fauna to move vertically or horizontally at small spatial scales to avoid stresses (Bengtsson, 2002; Nielsen et al., 2010; de Vries & Shade, 2013); and (d) historical effects on soil biodiversity might have already been reflected by contemporary environments as themselves are also shaped by paleoclimate (Svenning, Eiserhardt, Normand, Ordonez, & Sandel, 2015). Yet, we found that the variation in nematode diversity and community composition could be explained partly by the shared effects between contemporary and historical factors, highlighting the importance of interaction and complementarity among multiple processes/factors on soil biodiversity.

The climatic, soil, and historical factors only explained a small proportion of the total variation in nematode diversity and composition here, this could occur from some other potential and indirect explanatory variables, such as the characteristics of vegetation and soil microbes (providing the habitat as well as food for nematodes), have not been included in the current study (Decaëns, 2010; Wardle, 2006). Additionally, the strong heterogeneity of the soil environment along with the fluctuating nematode population dynamics can make the relationships obtained at local scales difficult to expand to large scales (Decaëns, 2010; Kerfahi et al., 2016; Nielsen et al., 2014; Paul, 2015). Furthermore, the diversity pattern of soil fauna is not only influenced by the deterministic processes discussed here but is also probably regulated by other factors, such as biotic interactions and stochastic processes (Caruso, Taormina, & Migliorini, 2012; Maaß, Migliorini, Rillig, & Caruso, 2014; Quist et al., 2019). We are also aware that the sample size was small here due to the logistic constraints at a large spatial scale. We suggest that appropriate caution should be applied in interpreting the results, and more work is really needed for further robust inferences and extrapolations. As there are numerous diversity and dissimilarity measures available for presence‐absence and abundance data (Magurran, 2004), only several were used and compared in current study. Given that different measures could be used to answer different ecological or biogeographic questions (Legendre, 2014), we expect to see more application of those complementary measures for better understanding of different aspects of soil biodiversity. Nevertheless, the variables and approaches adopted here are widely used in macroecology, which would contribute to a better comparison of current findings with other aboveground studies. We suggest further research should incorporate additional information (e.g., properties of vegetation, soil food web, and other historical factors) with large sample sizes to improve the mechanical understanding of historical effects on belowground communities across spatial scales in the future.

## 5 CONCLUSION

Although the independent effects of historical factors on soil nematodes were negligible, the shared effects due to the interactions between historical and other factors (e.g., climate and soil) were confirmed based on the current data. Our results highlight the potential of decomposing variation in soil biodiversity into different ecological processes, such as contemporary and historical processes, in the future.

## CONFLICT OF INTERESTS

The authors declare no conflict of interest.

## AUTHOR CONTRIBUTION

**Xianping Li:** Conceptualization (equal); Formal analysis (equal); Writing‐original draft (equal); Writing‐review & editing (equal). **Xiaoyun Chen:** Conceptualization (equal); Funding acquisition (equal); Investigation (equal); Resources (equal); Supervision (equal); Writing‐review & editing (equal). **Huimin Zhu:** Data curation (equal); Investigation (equal); Resources (equal). **Zhuhong Ren:** Data curation (equal); Investigation (equal); Resources (equal). **Jiaguo Jiao:** Conceptualization (equal); Investigation (equal); Resources (equal). **Feng Hu:** Conceptualization (equal); Funding acquisition (equal); Supervision (equal). **Manqiang Liu:** Conceptualization (equal); Funding acquisition (equal); Supervision (equal); Writing‐original draft (equal); Writing‐review & editing (equal).

## Supporting information

Supplementary Material

## Data Availability

Data are available at Zenodo (http://doi.org/10.5281/zenodo.3375277).
