# Supplementary material for: Effects of historical legacies on soil nematode communities are mediated by contemporary environmental conditions
Source: Ecol Evol. 2020 May 27;10(13):6732–40. doi: 10.1002/ece3.6406 (PMC7381565; doi:10.1002/ece3.6406)
Supplement: Supplementary file 1 — Supplementary Material [file ECE3-10-6732-s001.docx]

**Supporting information**

**Contents**

**Table S1.** The diversity and environmental attributes of the sampling sites.

**Table S2.** Correlations between climatic, soil, and historical variables.

**Table S3.** Genus lists for the sampling sites.

**Figure S1.** Distribution of the sampling sites.

**Figure S2.** Relationships between community dissimilarity and spatial distance.

**Figure S3.** Latitudinal variations in climatic, soil, and historical variables.

**Table S1. The summary of the diversity and environmental (i.e., climatic, soil, and historical) attributes of the sampling sites.** MAT: annual mean temperature; TS: temperature seasonality; MAP: annual precipitation; PS: precipitation seasonality; SOC: soil organic carbon; TN: total nitrogen; TA: temperature anomaly; PA: precipitation anomaly; TCV: temperature change velocity; PCV: precipitation change velocity. All the climatic and historical variables were extracted or calculated from WorldClim (http://www.worldclim.org/).

| **Site** | **Longitude** | **Latitude** | **Richness** | **Shannon’s diversity** |  | **Climate** | | | |  | **Soil** | | | |  | **Historical** | | | |
| --- | --- | --- | --- | --- | --- | --- | --- | --- | --- | --- | --- | --- | --- | --- | --- | --- | --- | --- | --- |
|  |  |  |  |  |  | **MAT** | **TS** | **MAP** | **PS** |  | **Taxonomy** | **SOC** | **TN** | **pH** |  | **TA** | **PA** | **TCV** | **PCV** |
| Site1 | 115.48 | 34.51 | 17 | 1.92 |  | 14.50 | 96.82 | 694.00 | 87.00 |  | Anthrepts | 7.69 | 0.55 | 8.35 |  | 4.90 | 58.00 | 21.65 | 2.37 |
| Site2 | 108.84 | 34.53 | 26 | 2.39 |  | 15.40 | 102.47 | 505.00 | 76.00 |  | Ustolls | 11.40 | 1.03 | 8.43 |  | 3.70 | 12.00 | 16.34 | 0.48 |
| Site3 | 98.29 | 27.68 | 32 | 2.91 |  | 17.20 | 47.30 | 2039.00 | 88.00 |  | Aqualfs | 74.25 | 5.51 | 4.41 |  | 3.30 | -386.00 | 0.25 | 0.26 |
| Site4 | 114.39 | 36.10 | 31 | 2.46 |  | 14.50 | 100.31 | 576.00 | 108.00 |  | Ustolls | 18.37 | 1.25 | 8.33 |  | 4.75 | 35.00 | 10.49 | 1.34 |
| Site5 | 116.41 | 39.90 | 22 | 2.24 |  | 12.00 | 106.82 | 609.00 | 131.00 |  | Ustolls | 7.97 | 0.67 | 8.56 |  | 5.00 | 40.00 | 13.44 | 1.22 |
| Site6 | 103.89 | 25.30 | 39 | 3.16 |  | 15.70 | 46.85 | 996.00 | 82.00 |  | Borolls | 36.63 | 2.65 | 6.82 |  | 2.75 | -270.50 | 3.66 | 4.35 |
| Site7 | 116.60 | 36.57 | 28 | 2.13 |  | 14.30 | 101.94 | 563.00 | 115.00 |  | Udolls | 16.73 | 1.70 | 8.20 |  | 5.15 | 50.00 | 14.25 | 1.32 |
| Site8 | 103.47 | 23.48 | 28 | 2.59 |  | 14.60 | 41.19 | 1257.00 | 85.00 |  | Udults | 60.52 | 4.36 | 7.25 |  | 2.15 | -33.00 | 0.59 | 0.12 |
| Site9 | 103.91 | 22.73 | 41 | 3.09 |  | 21.10 | 41.05 | 1517.00 | 79.00 |  | Udults | 37.13 | 2.99 | 5.62 |  | 2.05 | 186.50 | 0.38 | 1.26 |
| Site10 | 121.76 | 29.80 | 27 | 2.73 |  | 15.80 | 78.83 | 1430.00 | 41.00 |  | Udults | 14.28 | 1.38 | 7.59 |  | 8.20 | 0.00 | 3.35 | 0.00 |
| Site11 | 119.20 | 27.90 | 19 | 2.68 |  | 11.50 | 66.42 | 2132.00 | 49.00 |  | Ochrepts | 37.61 | 2.57 | 5.16 |  | 5.80 | 97.50 | 1.78 | 0.47 |
| Site12 | 118.20 | 25.63 | 34 | 3.22 |  | 16.60 | 56.06 | 1635.00 | 55.00 |  | Cryalfs | 20.50 | 1.58 | 5.36 |  | 4.90 | 165.50 | 0.68 | 0.53 |
| Site13 | 115.14 | 23.04 | 37 | 3.35 |  | 20.30 | 49.65 | 2028.00 | 70.00 |  | Xerolls | 22.12 | 1.41 | 5.01 |  | 2.85 | 306.50 | 0.61 | 0.79 |
| Site14 | 111.98 | 22.82 | 34 | 2.77 |  | 20.90 | 52.62 | 1663.00 | 69.00 |  | Borolls | 9.96 | 0.37 | 5.30 |  | 2.60 | -10.00 | 1.85 | 0.17 |
| Site15 | 112.98 | 24.75 | 34 | 3.06 |  | 16.90 | 65.64 | 1580.00 | 62.00 |  | Ochrepts | 39.67 | 3.14 | 4.67 |  | 2.40 | 161.00 | 0.38 | 0.55 |
| Site16 | 114.52 | 24.66 | 31 | 2.48 |  | 19.70 | 63.46 | 1739.00 | 63.00 |  | Ochrepts | 33.02 | 2.35 | 4.56 |  | 2.50 | 271.00 | 0.79 | 1.18 |

**Table S2. Correlations between climatic, soil, and historical variables.** The number of stars shows the significance (* *p* < 0.05; ** *p* < 0.01; *** *p* < 0.001), and significant values are shown in bold. Variable abbreviations as in Figure 1.

|  | **MAT** | **TS** | **MAP** | **PS** | **SOC** | **TN** | **pH** | **TA** | **PA** | **TCV** | **PCV** |
| --- | --- | --- | --- | --- | --- | --- | --- | --- | --- | --- | --- |
| **MAT** | 1.00 |  |  |  |  |  |  |  |  |  |  |
| **TS** | **-0.59*** | 1.00 |  |  |  |  |  |  |  |  |  |
| **MAP** | 0.46 | **-0.76***** | 1.00 |  |  |  |  |  |  |  |  |
| **PS** | -0.33 | 0.49 | **-0.67**** | 1.00 |  |  |  |  |  |  |  |
| **SOC** | 0.09 | **-0.66**** | **0.51*** | -0.12 | 1.00 |  |  |  |  |  |  |
| **TN** | 0.07 | **-0.60*** | 0.45 | -0.10 | **0.99***** | 1.00 |  |  |  |  |  |
| **pH** | **-0.59*** | **0.76***** | **-0.92***** | **0.57*** | **-0.53*** | -0.48 | 1.00 |  |  |  |  |
| **TA** | **-0.58*** | **0.57*** | -0.20 | -0.09 | -0.44 | -0.39 | 0.45 | 1.00 |  |  |  |
| **PA** | 0.27 | 0.09 | 0.14 | -0.24 | -0.42 | -0.42 | -0.16 | -0.04 | 1.00 |  |  |
| **TCV** | **-0.57*** | **0.87***** | **-0.85***** | 0.49 | **-0.72**** | **-0.70**** | **0.88***** | **0.50*** | -0.09 | 1.00 |  |
| **PCV** | -0.11 | 0.18 | -0.45 | 0.42 | -0.20 | -0.20 | 0.28 | -0.16 | -0.05 | 0.40 | 1.00 |

**Table S3. Genus lists of the nematodes for the sampling sites.**

| **Order** | **Family** | **Genus** | **Site1** | **Site2** | **Site3** | **Site4** | **Site5** | **Site6** | **Site7** | **Site8** | **Site9** | **Site10** | **Site11** | **Site12** | **Site13** | **Site14** | **Site15** | **Site16** |
| --- | --- | --- | --- | --- | --- | --- | --- | --- | --- | --- | --- | --- | --- | --- | --- | --- | --- | --- |
| Araeolaimida | Halaphanolaimidae | Aphanolaimus |  |  |  |  |  |  |  |  | **+** | **+** | **+** | **+** | **+** |  | **+** | **+** |
| Araeolaimida | Plectidae | Anaplectus |  |  |  |  | **+** |  |  |  | **+** | **+** |  |  |  | **+** |  |  |
| Araeolaimida | Plectidae | Chronogaster |  |  |  |  |  |  |  |  |  | **+** | **+** |  |  | **+** | **+** | **+** |
| Araeolaimida | Plectidae | Plectus | **+** |  | **+** | **+** | **+** | **+** |  |  | **+** |  | **+** | **+** | **+** | **+** | **+** | **+** |
| Araeolaimida | Plectidae | Tylocephalus |  |  |  | **+** |  |  |  | **+** |  |  |  |  | **+** |  |  |  |
| Chromadorida | Cyatholaimidae | Achromadora |  |  |  |  |  |  |  |  | **+** |  | **+** |  |  |  |  |  |
| Dorylaimida | Actinolaimidae | Paractinolaimus |  |  |  |  |  |  |  |  | **+** | **+** | **+** |  |  |  |  |  |
| Dorylaimida | Aporcelaimidae | Aporcelaimus | **+** | **+** | **+** | **+** | **+** | **+** | **+** | **+** |  |  | **+** | **+** | **+** | **+** | **+** |  |
| Dorylaimida | Belondiridae | Axonchium |  | **+** |  |  |  | **+** | **+** | **+** |  |  |  | **+** |  | **+** |  |  |
| Dorylaimida | Belondiridae | Belondira |  |  |  |  |  | **+** |  |  |  |  |  |  |  |  |  |  |
| Dorylaimida | Belondiridae | Oxydirus |  |  | **+** |  |  |  |  |  | **+** | **+** | **+** | **+** |  | **+** | **+** |  |
| Dorylaimida | Dorylaimidae | Mesodorylaimus |  | **+** | **+** |  | **+** | **+** | **+** |  |  | **+** | **+** | **+** | **+** | **+** |  | **+** |
| Dorylaimida | Leptonchidae | Leptonchus |  |  |  |  |  |  | **+** |  |  |  |  |  | **+** |  |  |  |
| Dorylaimida | Longidoridae | Longidorus | **+** | **+** | **+** |  | **+** | **+** | **+** | **+** |  |  |  |  | **+** | **+** |  |  |
| Dorylaimida | Longidoridae | Xiphinema |  |  |  |  |  | **+** |  |  |  | **+** |  |  | **+** |  | **+** | **+** |
| Dorylaimida | Nordiidae | Oriverutus | **+** |  | **+** |  |  | **+** | **+** | **+** | **+** |  |  | **+** | **+** | **+** | **+** | **+** |
| Dorylaimida | Nordiidae | Pungentus |  |  | **+** | **+** |  | **+** | **+** | **+** | **+** |  |  | **+** |  |  |  | **+** |
| Dorylaimida | Nygolaimidae | Nygolaimus |  |  |  |  |  |  |  | **+** | **+** | **+** |  |  | **+** | **+** | **+** |  |
| Dorylaimida | Qudsianematidae | Chrysonemoides |  |  | **+** | **+** |  |  |  | **+** | **+** |  |  |  |  | **+** | **+** | **+** |
| Dorylaimida | Qudsianematidae | Eudorylaimus | **+** | **+** | **+** | **+** | **+** | **+** | **+** | **+** | **+** | **+** |  | **+** | **+** | **+** | **+** | **+** |
| Dorylaimida | Qudsianematidae | Microdorylaimus | **+** | **+** | **+** | **+** | **+** | **+** | **+** | **+** | **+** |  |  | **+** | **+** | **+** | **+** | **+** |
| Dorylaimida | Qudsianematidae | Prodorylaimus |  |  | **+** | **+** |  | **+** |  | **+** | **+** | **+** | **+** | **+** | **+** | **+** | **+** | **+** |
| Dorylaimida | Tylencholaimidae | Tylencholaimus |  |  |  |  |  | **+** |  |  | **+** | **+** |  | **+** |  | **+** | **+** | **+** |
| Enoplida | Alaimidae | Alaimus |  | **+** | **+** | **+** |  | **+** | **+** |  | **+** | **+** |  | **+** | **+** | **+** | **+** | **+** |
| Monhysterida | Monhysteridae | Monhystrella |  | **+** | **+** | **+** |  | **+** | **+** |  |  |  |  | **+** |  |  |  | **+** |
| Mononchida | Anatonchidae | Miconchus |  |  | **+** |  |  |  |  |  | **+** |  |  | **+** | **+** |  | **+** |  |
| Mononchida | Mononchidae | Iotonchus |  |  | **+** |  |  |  |  | **+** |  |  |  |  |  |  | **+** |  |
| Mononchida | Mononchidae | Mononchus |  | **+** | **+** |  |  | **+** | **+** |  | **+** | **+** | **+** | **+** | **+** |  | **+** | **+** |
| Mononchida | Mononchidae | Prionchulus |  |  |  |  |  | **+** |  |  |  |  | **+** | **+** | **+** |  | **+** |  |
| Mononchida | Mylonchulidae | Mylonchulus |  |  |  |  |  | **+** |  |  |  |  |  |  |  |  |  |  |
| Rhabditida | Cephalobidae | Acrobeles | **+** |  |  | **+** |  |  |  | **+** |  |  |  |  |  |  |  |  |
| Rhabditida | Cephalobidae | Acrobeloides | **+** | **+** | **+** | **+** | **+** | **+** | **+** | **+** | **+** | **+** | **+** | **+** | **+** | **+** | **+** | **+** |
| Rhabditida | Cephalobidae | Cephalobus |  | **+** |  | **+** |  |  | **+** |  | **+** |  |  |  |  |  |  |  |
| Rhabditida | Cephalobidae | Cervidellus |  |  |  |  |  |  |  | **+** |  |  |  |  |  |  |  |  |
| Rhabditida | Cephalobidae | Eucephalobus | **+** | **+** | **+** | **+** | **+** | **+** | **+** | **+** | **+** | **+** |  | **+** | **+** | **+** |  | **+** |
| Rhabditida | Diploscapteridae | Diploscapter |  |  |  |  | **+** |  | **+** |  |  |  |  |  |  |  |  |  |
| Rhabditida | Panagrolaimidae | Panagrolaimus |  | **+** |  |  |  |  | **+** |  | **+** |  |  |  |  |  |  | **+** |
| Rhabditida | Rhabditidae | Mesorhabditis |  | **+** | **+** | **+** | **+** | **+** | **+** |  | **+** | **+** |  | **+** |  | **+** |  | **+** |
| Rhabditida | Rhabditidae | Protorhabditis |  | **+** | **+** | **+** | **+** | **+** | **+** |  | **+** | **+** |  | **+** | **+** | **+** | **+** | **+** |
| Rhabditida | Rhabditidae | Rhabditis |  | **+** |  |  |  |  |  |  | **+** |  |  | **+** |  |  |  |  |
| Rhabditida | Teratocephalidae | Metateratocephalus | |  |  |  |  | **+** |  |  |  |  |  |  |  |  |  |  |
| Triplonchida | Diphtherophoridae | Diphtherophora |  | **+** |  | **+** |  |  |  |  |  |  |  | **+** | **+** | **+** |  | **+** |
| Triplonchida | Prismatolaimidae | Prismatolaimus |  |  | **+** |  |  | **+** |  |  | **+** |  | **+** | **+** | **+** |  | **+** | **+** |
| Triplonchida | Tripylidae | Tripyla |  |  |  | **+** |  |  |  |  | **+** | **+** |  | **+** |  |  | **+** | **+** |
| Tylenchida | Anguinidae | Ditylenchus |  | **+** | **+** | **+** | **+** | **+** | **+** | **+** | **+** |  | **+** | **+** | **+** | **+** | **+** |  |
| Tylenchida | Aphelenchidae | Aphelenchus | **+** | **+** |  | **+** | **+** | **+** | **+** | **+** | **+** | **+** |  |  | **+** | **+** | **+** |  |
| Tylenchida | Aphelenchoididae | Aphelenchoides |  |  | **+** | **+** | **+** | **+** | **+** | **+** | **+** |  | **+** | **+** | **+** | **+** | **+** | **+** |
| Tylenchida | Criconematidae | Criconema |  |  | **+** |  |  |  |  | **+** | **+** |  |  | **+** | **+** | **+** | **+** |  |
| Tylenchida | Criconematidae | Criconemoides |  |  |  |  | **+** | **+** |  |  | **+** |  |  |  |  |  |  |  |
| Tylenchida | Dolichodoridae | Dolichodorus |  |  |  |  |  |  |  |  |  |  |  |  | **+** |  |  | **+** |
| Tylenchida | Hemicycliophoridae | Hemicycliophora |  |  |  |  |  |  |  |  |  |  |  |  | **+** |  |  |  |
| Tylenchida | Hoplolaimidae | Helicotylenchus | **+** | **+** | **+** | **+** | **+** | **+** | **+** | **+** | **+** | **+** |  | **+** | **+** | **+** | **+** | **+** |
| Tylenchida | Hoplolaimidae | Rotylenchulus | **+** | **+** | **+** | **+** | **+** | **+** | **+** | **+** | **+** | **+** |  |  | **+** | **+** | **+** | **+** |
| Tylenchida | Meloidogynidae | Meloidogyne | **+** |  | **+** | **+** |  | **+** | **+** | **+** | **+** | **+** |  |  | **+** | **+** |  | **+** |
| Tylenchida | Pratylenchidae | Pratylenchus | **+** | **+** | **+** | **+** | **+** | **+** |  | **+** | **+** |  |  |  |  | **+** | **+** |  |
| Tylenchida | Telotylenchidae | Tylenchorhynchus | **+** | **+** | **+** | **+** | **+** | **+** | **+** | **+** | **+** | **+** | **+** |  | **+** | **+** |  | **+** |
| Tylenchida | Tylenchidae | Basiria |  | **+** | **+** | **+** | **+** | **+** | **+** |  | **+** | **+** |  | **+** | **+** | **+** | **+** | **+** |
| Tylenchida | Tylenchidae | Boleodorus | **+** |  |  | **+** |  | **+** |  |  | **+** | **+** |  |  |  |  |  |  |
| Tylenchida | Tylenchidae | Filenchus |  |  | **+** | **+** |  |  |  | **+** | **+** |  | **+** | **+** | **+** | **+** | **+** | **+** |
| Tylenchida | Tylenchidae | Lelenchus |  |  |  |  |  | **+** |  |  |  |  |  |  |  |  | **+** |  |
| Tylenchida | Tylenchidae | Malenchus | **+** | **+** |  | **+** |  | **+** |  |  | **+** | **+** | **+** | **+** | **+** | **+** | **+** |  |
| Tylenchida | Tylenchidae | Psilenchus |  |  |  |  |  | **+** | **+** |  |  |  |  | **+** | **+** |  |  |  |
| Tylenchida | Tylenchidae | Tylenchus |  | **+** | **+** | **+** | **+** | **+** |  | **+** | **+** | **+** | **+** | **+** | **+** | **+** | **+** |  |
| Tylenchida | Tylenchulidae | Paratylenchus |  |  |  |  |  |  |  | **+** |  |  |  |  |  |  |  |  |


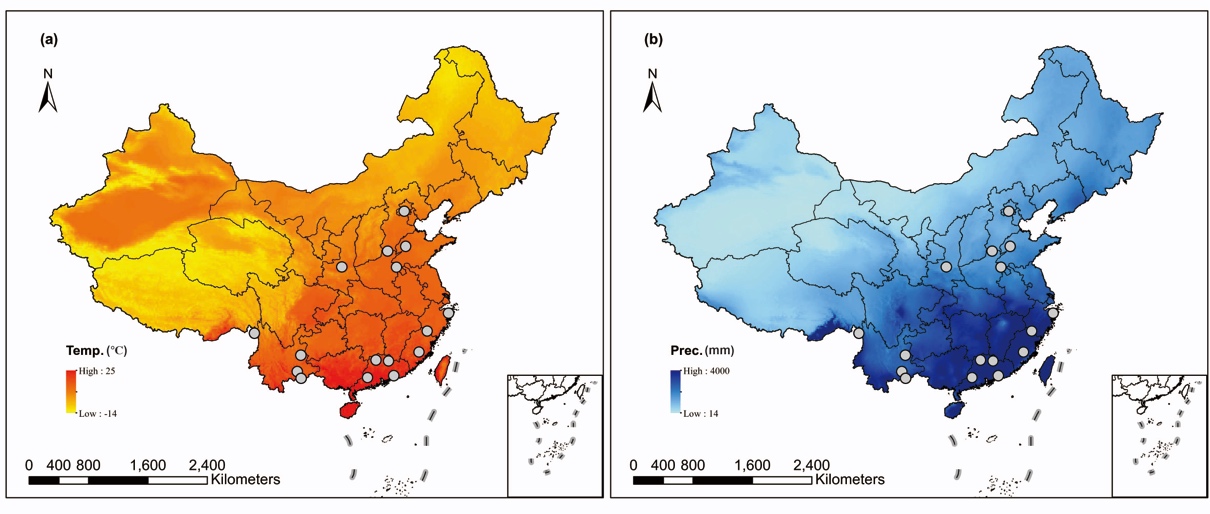


**Figure S1. Distribution of the sampling sites across temperature (a) and precipitation (b) gradients in mainland China.**


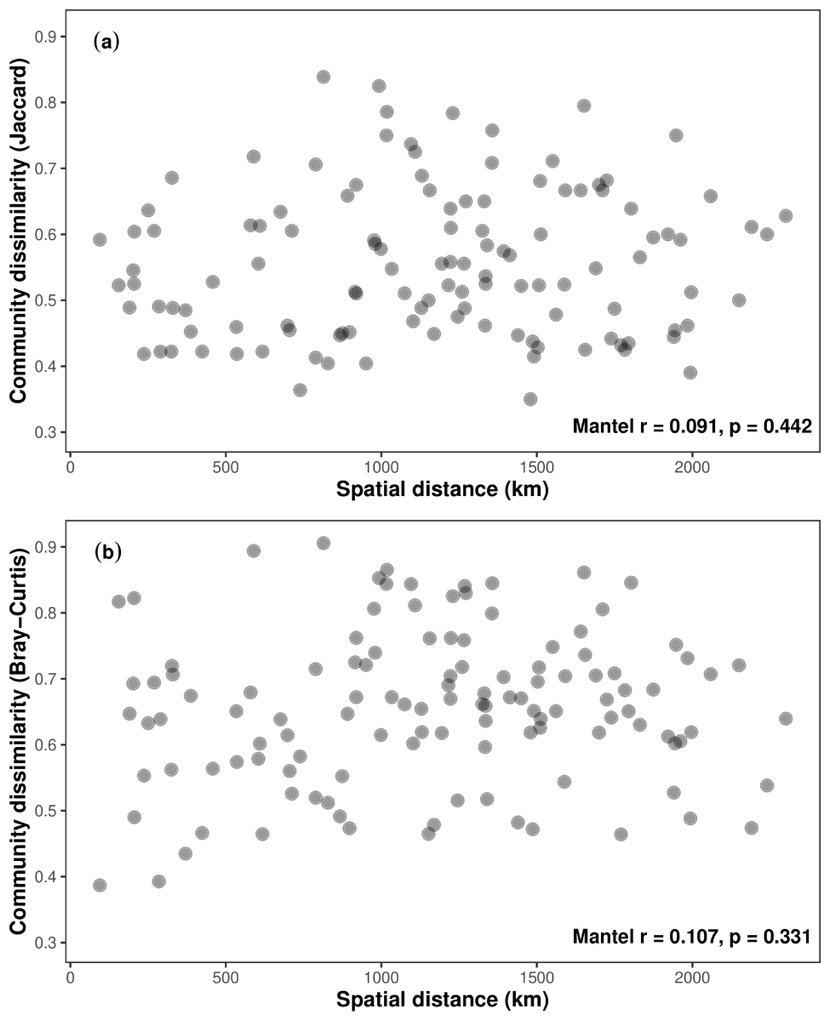


**Figure S2. Relationships between soil nematode community dissimilarity and spatial distance.** a, Jaccard dissimilarity; b, Bray-Curtis dissimilarity.


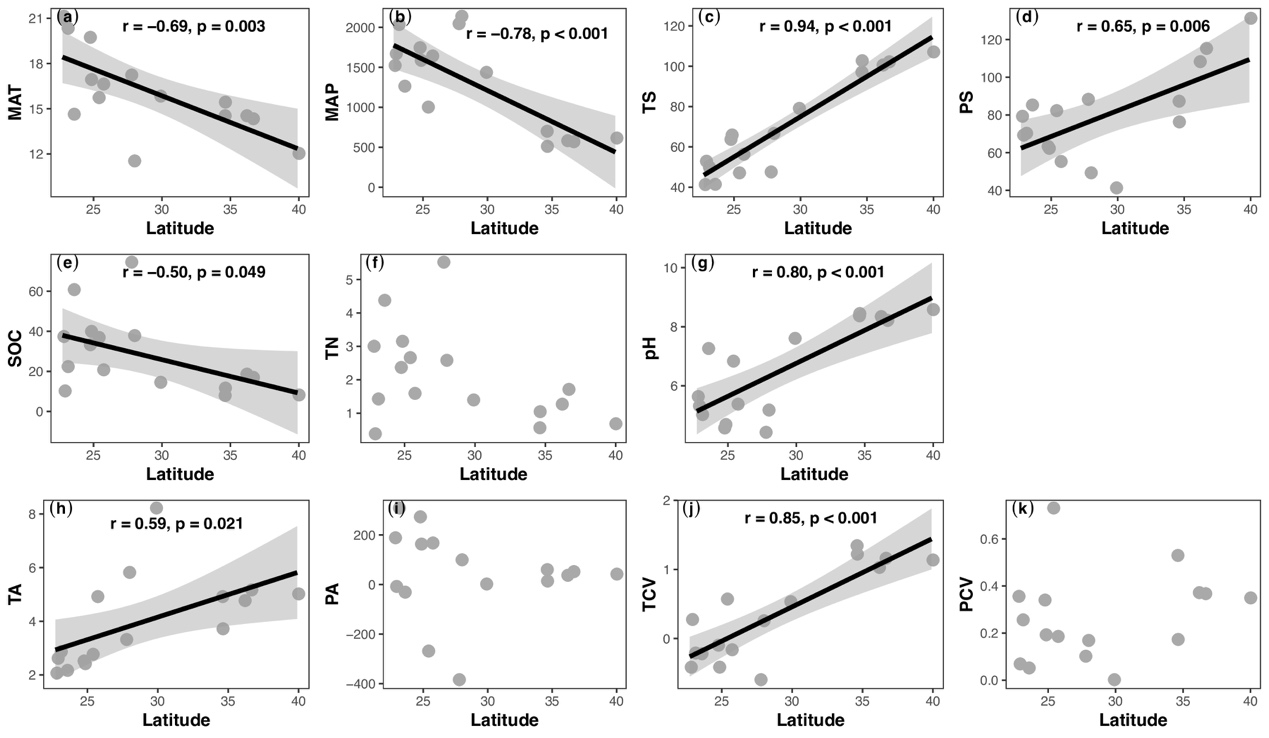


**Figure S3. Latitudinal variations in climatic, soil, and historical variables.** Only those variables shown significant trends were regressed against latitude. MAT: annual mean temperature; TS: temperature seasonality; MAP: annual precipitation; PS: precipitation seasonality; SOC: soil organic carbon; TN: total nitrogen; TA: temperature anomaly; PA: precipitation anomaly; TCV: temperature change velocity; PCV: precipitation change velocity.
